# Supplementary material for: Switching action modes of miR408-5p mediates auxin signaling in rice
Source: Nat Commun. 2024 Mar 21;15:2525. doi: 10.1038/s41467-024-46765-z (PMC10958043; doi:10.1038/s41467-024-46765-z)
Supplement: Supplementary file 1 — Supplementary Information [file 41467_2024_46765_MOESM1_ESM.pdf]

Supplementary Information

Switching action modes of miR408-5p mediates auxin signaling in rice

Fuxi Rong[1,2,6], Yusong Lv[1,2,6], Pingchuan Deng[1,3,6], Xia Wu[1], Yaqi Zhang[2], Erkui Yue[1], Yuxin Shen[1], Sajid Muhammad [1], Fangrui Ni[1], Hongwu Bian[4], Xiangjin Wei[5], Weijun Zhou[1], Peisong Hu[5], Liang Wu[1,2,*]

1. National Key Laboratory of Rice Biology and Zhejiang Provincial Key Laboratory of Crop Germplasm Resources, College of Agriculture and Biotechnology, Zhejiang University, Hangzhou, Zhejiang, 310058, China
2. Hainan Yazhou Bay Seed Laboratory, Hainan Institute, Zhejiang University, Sanya, Hainan, 572000, China
3. State Key Laboratory of Crop Stress Biology in Arid Areas, College of Agronomy, Northwest A&F University, Yangling, Shaanxi, 712100, China.
4. Institute of Genetics and Regenerative Biology, Key Laboratory for Cell and Gene Engineering of Zhejiang Province, College of Life Sciences, Zhejiang University, Hangzhou, 310058, China
5. National Key Laboratory of Rice Biology, China National Center for Rice Improvement, China National Rice Research Institute, Hangzhou, Zhejiang, 310006, China.
6. These authors contributed equally to this work.

* Correspondence should be addressed to: L.W. (liangwu@zju.edu.cn)

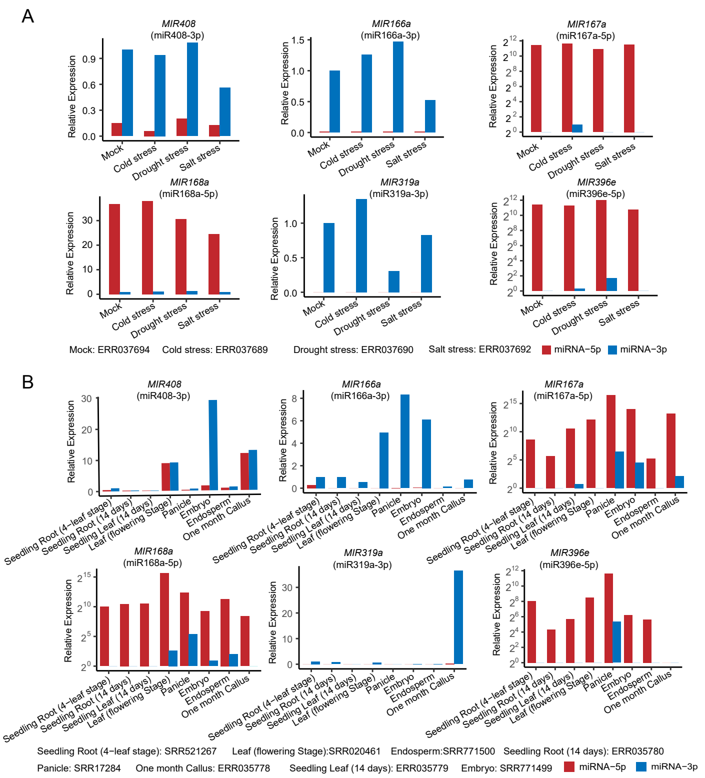

Supplementary Figure 1 miR408-5p is an abundant miRNA in rice

(A) Relative expressions of two mature miRNAs on different arms of one miRNA precursor in rice under diverse stresses. The expression matrix for each miRNA was retrieved from PmiRExAt database. The mature miRNA in bracket below the precursor denotes the highly conserved miRNA in different plants. miRNA-3p in mock was set as a control.

(B) Relative expressions of two miRNAs on each arm of one miRNA hairpin structure precursor in different tissues. The expression matrix for each miRNA was retrieved from PmiRExAt database. The mature miRNA in brackets below the precursor denote the highly conserved miRNA in different plants. miRNA-3p in seedling Root (4−leaf stage) was set as a control.

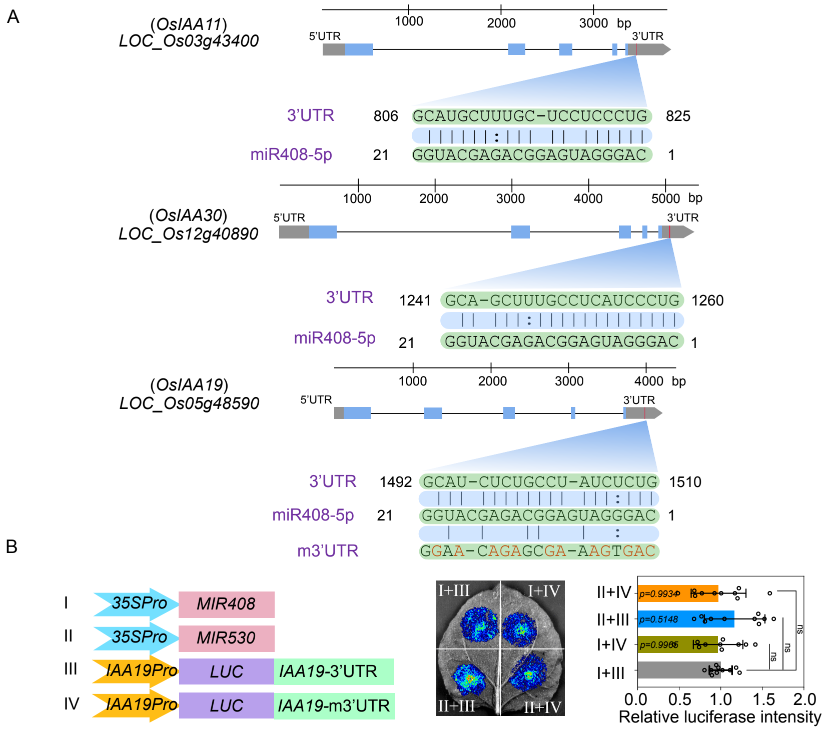

Supplementary Figure 2 miR408-5p regulates *IAA30* rather than *IAA19* in rice

(A) Sequence alignment of miR408-5p with target site of rice *IAA* orthologs which were predicted as miR408-5p targets by the PsRobot software.

(B) *IAA19* is not the target of miR408-5p tested through transient expression analysis in *N.benthamiana* leaves. Left: The constructs in *A. tumefaciens* transiently introduced into *N. benthamiana* leaves. Middle: Representative photograph of firefly luciferase fluorescence signals when the indicated construct combinations were introduced in *N. benthamiana* leaves. Right: Relative reporter activity in *N. benthamiana* leaves expressing the indicated construct combinations. Error bars indicate SD (Multiple comparisons test; ns, no significance). Source data underlying Supplementary Fig. 2B are provided as a Source Data file.

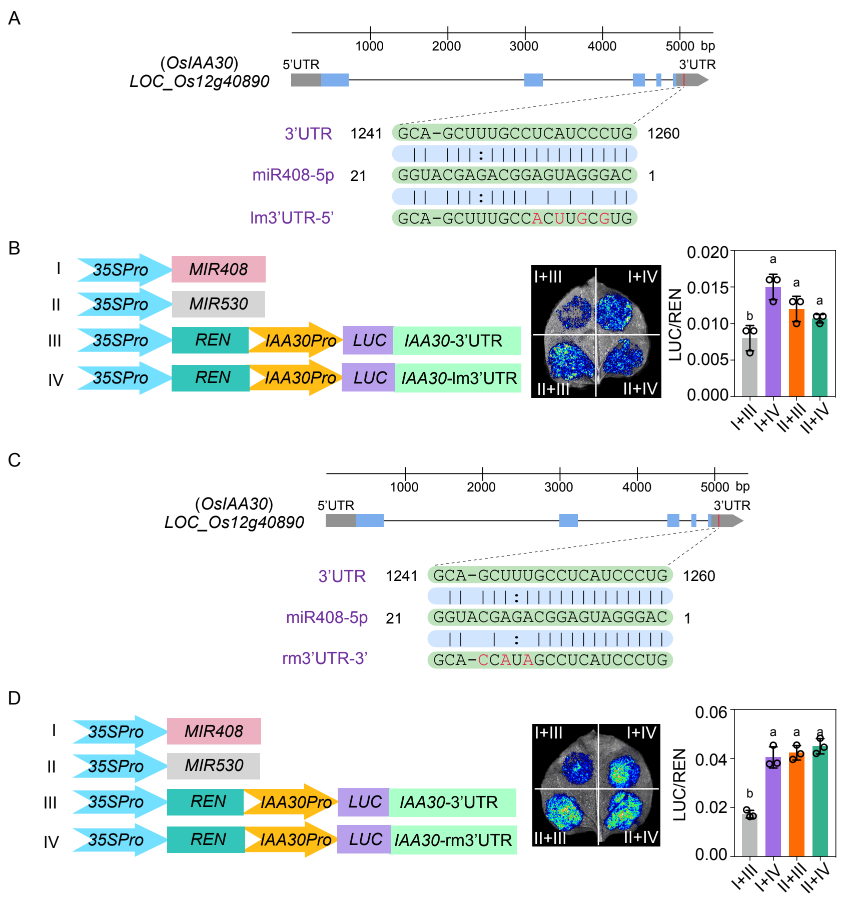

Supplementary Figure 3 Regulation of *IAA30* by miR408-5p in rice

(A) Gene structure of *IAA30* and alignments of miR408-5p with target sites in original *IAA30* 3' UTR (3' UTR) and the indicated mutated 3' UTR (lm3' UTR), which mismatches with the sequences at 5' left arm of miR408-5p.

(B) Regulation examination of 3' UTR and lm3' UTR of *IAA30* by miR408-5p through transient expression analysis in *N. benthamiana* leaves. Left: The constructs in *A. tumefaciens* transiently introduced in *N. benthamiana* leaves. Middle: Representative photograph of firefly luciferase fluorescence signals when the indicated construct combinations were introduced in *N. benthamiana* leaves. Right: Relative reporter activity in *N. benthamiana* leaves expressing the indicated construct combinations. Error bars indicate SD (Tukey's honestly significant difference, $P < 0.05$).

(C) Gene structure of *IAA30* and alignments of miR408-5p with target sites in *IAA30* 3' UTR and the indicated mutated 3' UTR (rm3' UTR), which mismatches with the sequences at 3' right arm of miR408-5p.

(D) Regulation examination of 3' UTR and rm3' UTR of *IAA30* as miR408-5p target through

transient expression analysis in *N. benthamiana* leaves. Left: The constructs in *A. tumefaciens* transiently introduced in *N. benthamiana* leaves. Middle: Representative photograph of firefly luciferase fluorescence signals when the indicated construct combinations were introduced in *N. benthamiana* leaves. Right: Relative reporter activity in *N. benthamiana* leaves expressing the indicated construct combinations. Error bars indicate SD (Tukey's honestly significant difference, $P < 0.05$). Source data underlying Supplementary Fig. 3B and D are provided as a Source Data file.

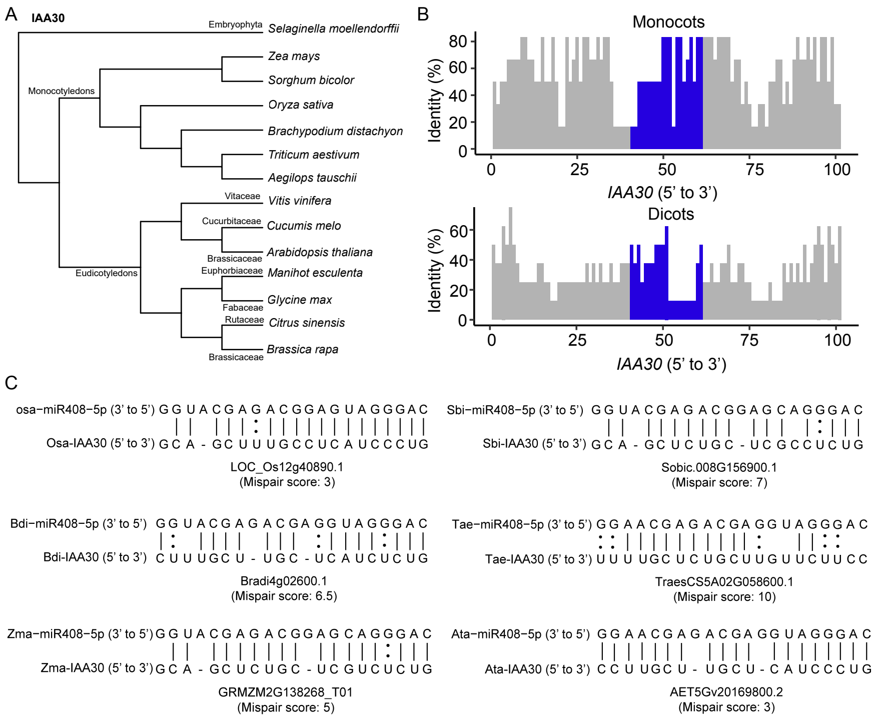

Supplementary Figure 4 Potential regulation of *IAA30* by miR408-5p in rice and some other monocots

(A) The evolutionary relationship of IAA30 in diverse plants. An un-rooted tree was constructed for IAA30 from 14 representative plant species using RAXML with maximum-likelihood (ML) method.

(B) Conservation analysis of 100bp region around the miR408-5p target sites in 3'UTR *IAA30* orthologs in typical monocot and dicot species. Blue bars denote the targeting region between miR408-5p and *IAA30*.

(C) Alignment of the possible binding sites between miR408-5p and *IAA30* orthologs in six representative monocotyledon species including rice, *Brachypodium*, maize, sorghum, wheat and *Aegilops tauschii*. *IAA30* ortholog in *Aegilops tauschii* may be targeted by miR408-5p because the mispairing score is below 4.

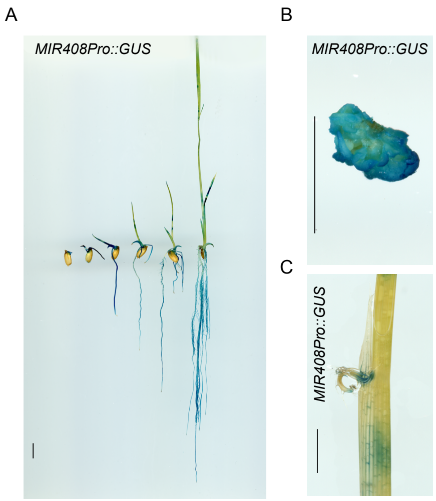

Supplementary Figure 5 *MIR408* is universally expressed in different tissues in rice

(A) GUS staining of *MIR408* promoter-driven GUS transgenic plants with different stages. Bar = 1cm

(B) GUS staining in callus of *MIR408* promoter-driven GUS transgenic rice. Bar = 1cm

(C) GUS staining in leaf joint of *MIR408* promoter-driven GUS transgenic rice. Bar = 1cm

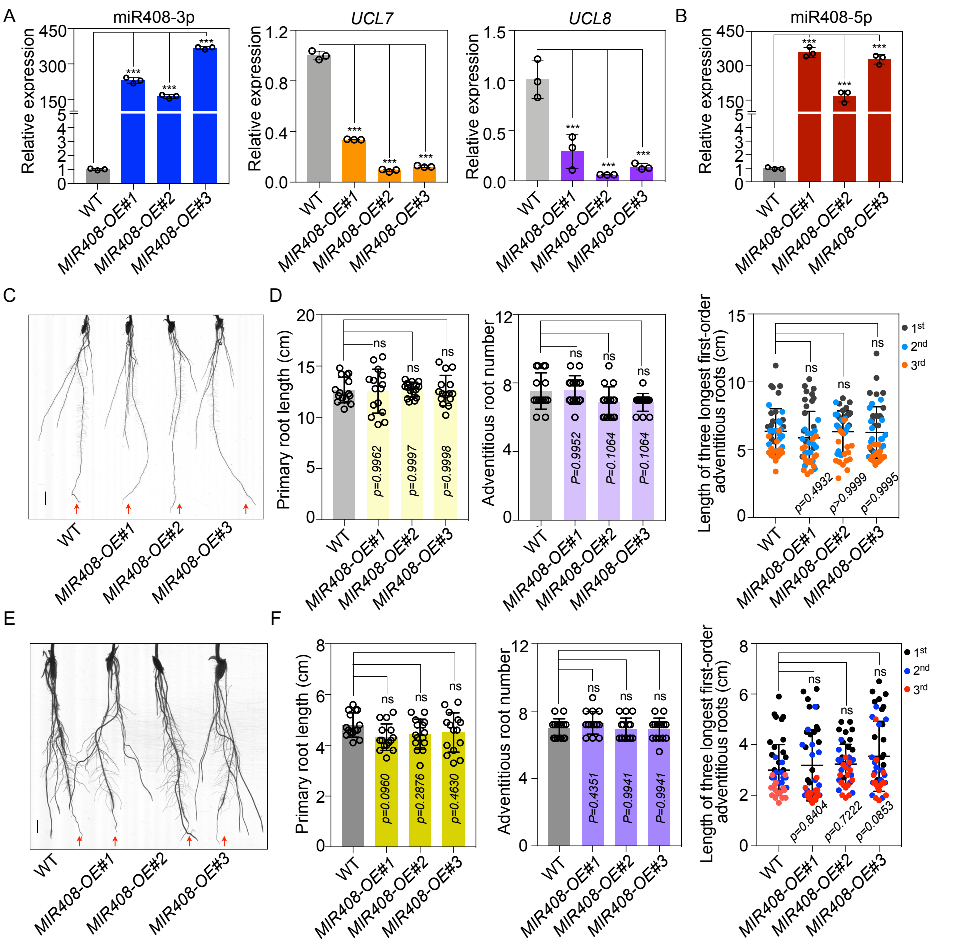

Supplementary Figure 6 *MIR408* overexpression has no obvious influence on auxin response in rice

(A) The expressions of miR403-3p, *UCL7* and *UCL8* in WT and *MIR408-OE* transgenic rice plants. The value of indicated gene transcripts in WT was arbitrarily designated as 1. Error bars represent SD for three replicates. Statistical analysis was carried out by using Multiple comparisons test (**P < 0.01, ***P < 0.001)

(B) The expression of miR408-5p in WT and *MIR408-OE* transgenic rice plants.

(C) Representative phenotype of seedling roots of 2-week-old WT and *MIR408-OE* transgenic plants without IAA treatment. Red arrow indicates primary roots. Bar = 1cm

(D) Primary root length, adventitious root number and the length of three longest first-order crown roots in 2-week-old WT and *MIR408-OE* transgenic plants without IAA treatment.

(E) Representative phenotype of seedling roots of 2-week-old WT and *MIR408-OE* transgenic plants under 10μM IAA treatment. Red arrow indicates primary roots. Bar = 1cm

(F) Primary root length, adventitious root number and the length of three longest first-order crown

roots in 2-week-old WT and *MIR408-OE* transgenic plants under 10μM IAA treatment. Source data underlying Supplementary Fig. 6A, B, D, and F are provided as a Source Data file.

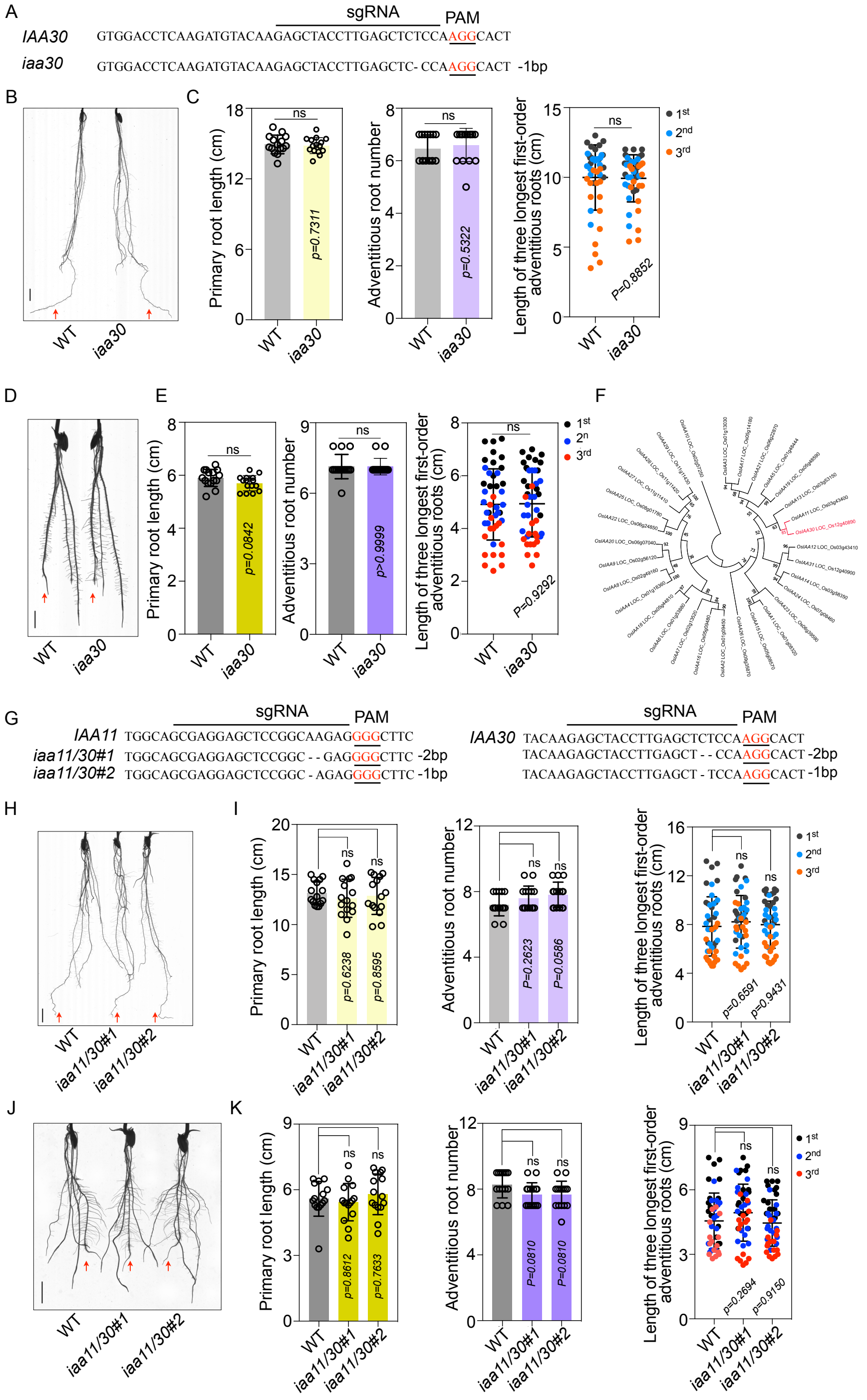

Supplementary Figure 7 IAA30 plays redundant roles with other IAA paralogs in auxin signaling in rice

(A) CRISPR/Cas9-mediated target mutagenesis of *IAA30* in rice. The red color sequences mean protospacer adjacent motifs.

(B) Representative phenotype of seedling roots of 2-week-old WT and *iaa30* mutants without auxin treatment. Red arrow indicates primary roots. Bar = 1cm

(C) Primary root length, adventitious root number and the length of three longest first-order crown roots in 2-week-old WT and *iaa30* mutants without auxin treatment.

(D) Representative phenotype of seedling roots of 2-week-old WT and *iaa30* mutants under 10μM IAA treatment. Red arrow indicates primary roots. Bar = 1cm

(E) Primary root length, adventitious root number and the length of three longest first-order crown roots in 2-week-old WT and *iaa30* mutants under 10μM IAA treatment.

(F) A phylogenetic tree of IAA family proteins in rice.

(G) CRISPR/Cas9-mediated simultaneous mutagenesis of *IAA11* and *IAA30* in rice. The red color sequences mean protospacer adjacent motifs.

(H) Representative phenotype of seedling roots of 2-week-old WT and *iaa11/30* double mutants without auxin treatment. Red arrow indicates primary roots. Bar = 1cm

(I) Primary root length, adventitious root number and the length of three longest first-order crown roots in 2-week-old WT and *iaa11/30* double mutants without auxin treatment.

(J) Representative phenotype of seedling roots of 2-week-old WT and *iaa11/30* double mutants under 10μM IAA treatment. Bar = 1cm

(K) Primary root length, adventitious root number and the length of three longest first-order crown roots in 2-week-old WT and *iaa11/30* double mutants under 10μM IAA treatment. Source data underlying Supplementary Fig. 7C, E, I, and K are provided as a Source Data file.

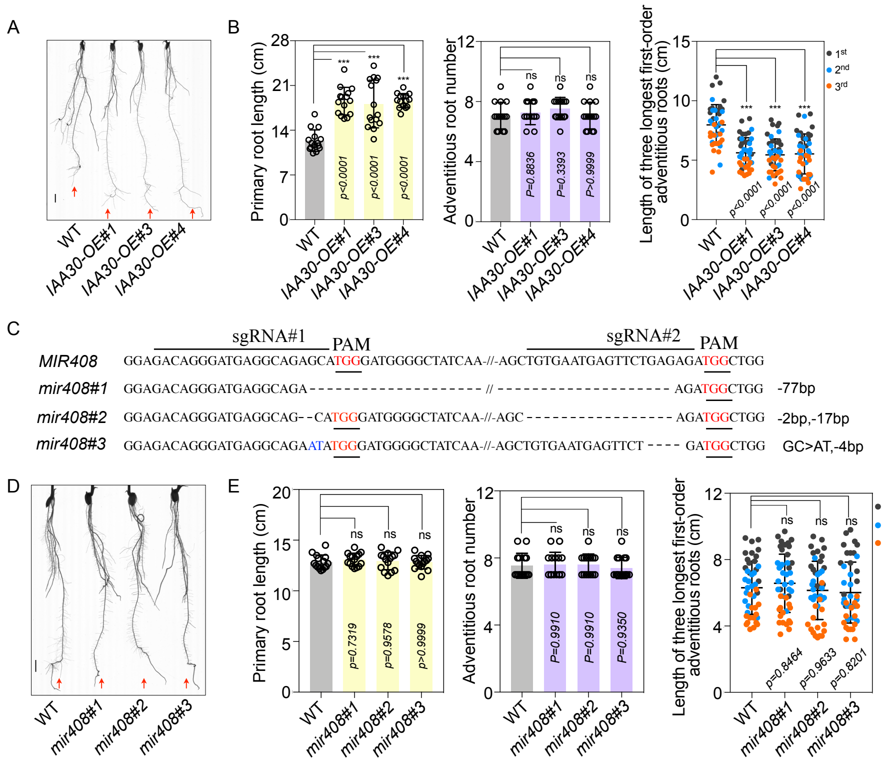

Supplementary Figure 8 The root performances of *IAA30-OE* and *mir408* plants without IAA treatment

(A) Representative phenotype of seedling roots of 2-week-old WT and *IAA30-OE* plants without auxin treatment. Bar = 1cm

(B) Primary root length, adventitious root number and the length of three longest first-order crown roots in 2-week-old WT and *IAA30-OE* plants without auxin treatment.

(C) CRISPR/Cas9-mediated target mutagenesis of *MIR408* to generate *mir408* mutants in rice. The red color sequences mean protospacer adjacent motifs.

(D) Representative phenotype of seedling roots of 2-week-old *mir408* mutants without auxin treatment. Red arrow indicates primary roots. Bar = 1cm

(E) Primary root length, adventitious root number and the length of three longest first-order crown roots in 2-week-old *mir408* mutants without auxin treatment. Source data underlying Supplementary Fig. 8 B and E are provided as a Source Data file.

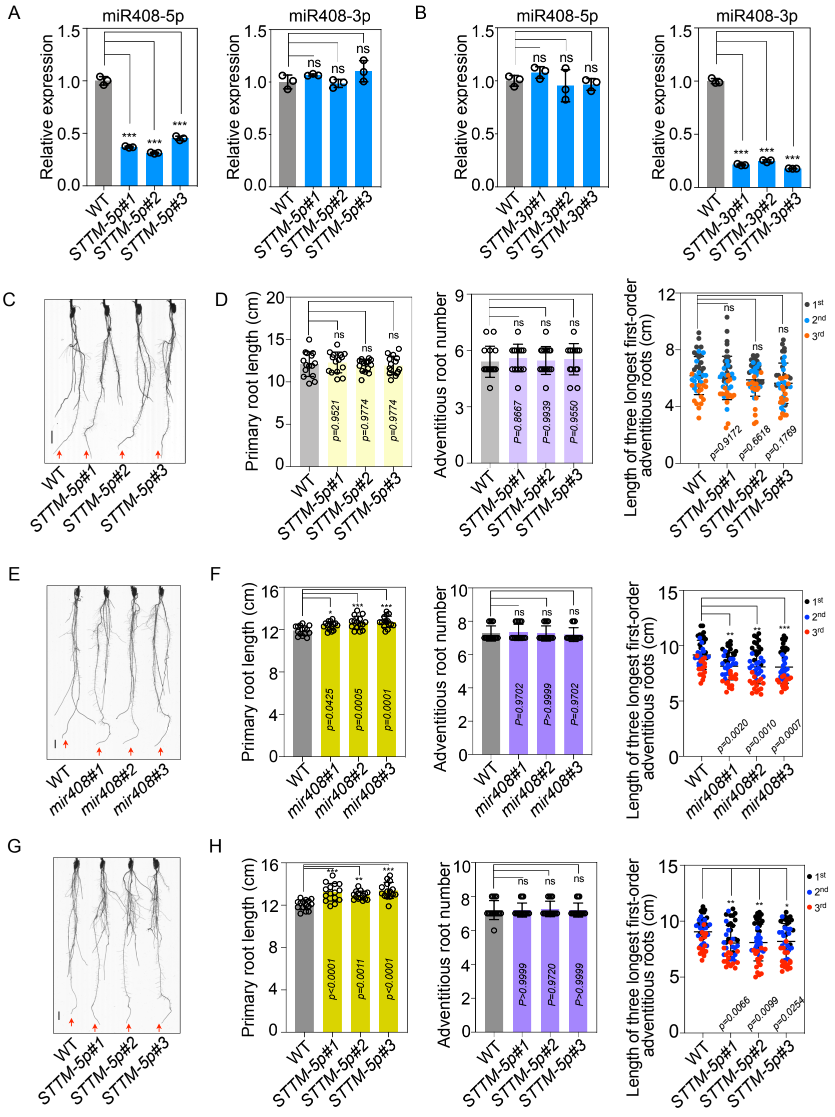

Supplementary Figure 9 miR408-5p is essential for *MIR408*-mediated auxin response in rice

(A) Relative expressions of miR408-5p and miR408-3p in WT and *STTM-5p* plants.

(B) Relative expressions of miR408-5p and miR408-3p in WT and *STTM-3p* plants.

(C) Representative phenotype of seedling roots of 2-week-old WT and *STTM-5p* plants without auxin treatment. Bar = 1cm

(D) Primary root length, adventitious root number and the length of three longest first-order crown roots in 2-week-old WT and *STTM-5p* plants without auxin treatment.

(E) Representative phenotype of seedling roots of 2-week-old WT and *mir408* mutants under 1μM IAA treatment. Bar = 1cm

(F) Primary root length, adventitious root number and the length of three longest first-order crown roots in 2-week-old WT and *mir408* mutants under 1μM IAA treatment.

(G) Representative phenotype of seedling roots of 2-week-old WT and *STTM-5p* plants under 1μM IAA treatment. Bar = 1cm

(H) Primary root length, adventitious root number and the length of three longest first-order crown roots in 2-week-old WT and *STTM-5p* plants under 1μM IAA treatment. Source data underlying Supplementary Fig. 9A, B, D, F, and H are provided as a Source Data file.

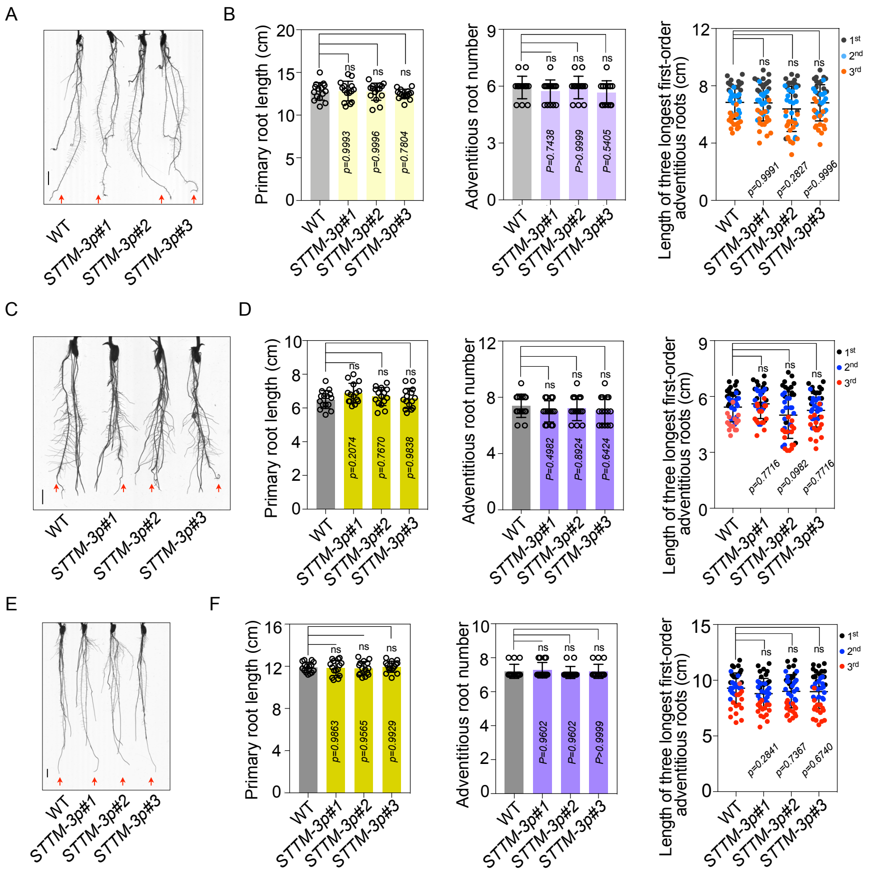

Supplementary Figure 10 miR408-3p may not be required for *MIR408*-mediated auxin response in rice

(A) Representative phenotype of seedling roots of 2-week-old WT and *STTM-3p* plants without auxin treatment. Bar = 1cm

(B) Primary root length, adventitious root number and the length of three longest first-order crown roots in 2-week-old WT and *STTM-3p* plants without auxin treatment.

(C) Representative phenotype of seedling roots of 2-week-old WT and *STTM-3p* plants under 10μM IAA treatment. Bar = 1cm

(D) Primary root length, adventitious root number and the length of three longest first-order crown roots in 2-week-old WT and *STTM-3p* plants under 10μM IAA treatment.

(E) Representative phenotype of seedling roots of 2-week-old WT and *STTM-3p* plants under 1μM IAA treatment. Bar = 1cm

(F) Primary root length, adventitious root number and the length of three longest first-order crown roots in 2-week-old WT and *STTM-3p* plants under 1 μM IAA treatment. Source data underlying Supplementary Fig. 10B, D, and F are provided as a Source Data file.

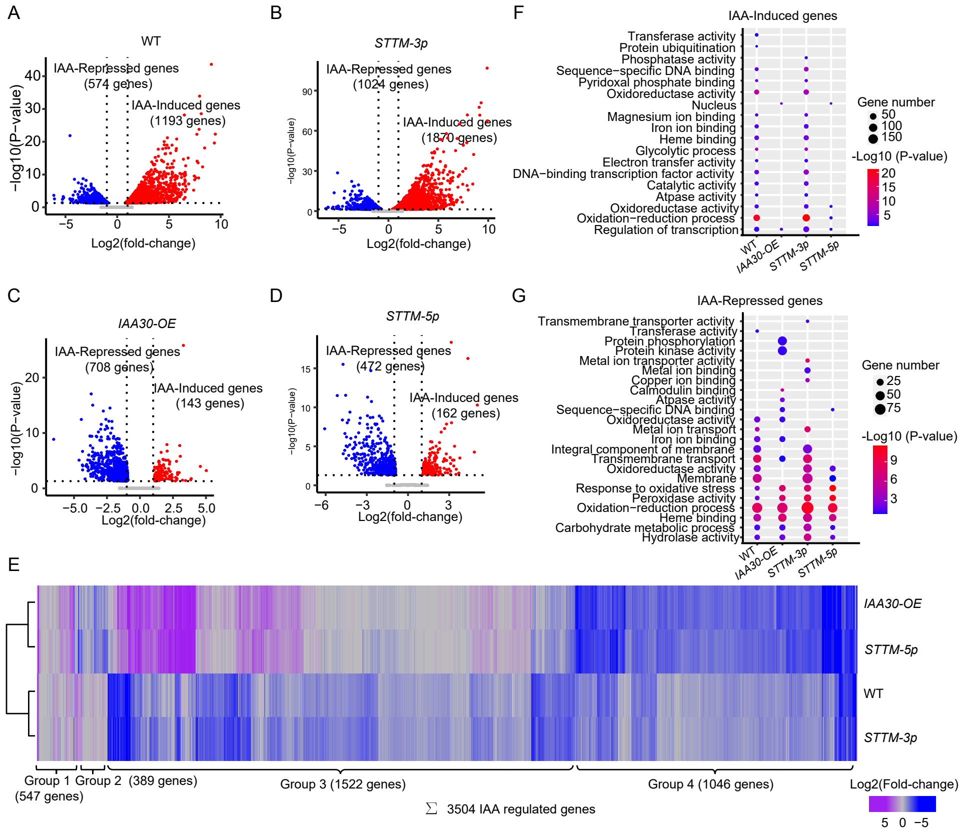

Supplementary Figure 11 RNA-seq analysis of gene expressions in WT, *STTM-5p*, *STTM-3p* and *IAA30-OE* plants with or without an auxin treatment

(A)-(D) The scatter plot of log2-fold change values for auxin regulated genes in WT (A), *STTM-3p* (B), *IAA30-OE* (C) and *STTM-5p* (D) plants.

(E) Heat map of 3504 genes responsive to auxin in *STTM-5p*, *STTM-3p*, *IAA30-OE* and WT. The scale bar indicates fold change of genes that were affected by auxin treatment compared with mock. Group 1 are genes induced in *STTM-5p*, *STTM-3p*, *IAA30-OE* and WT by 4h IAA treatment. Group 2 are genes induced only in WT and *STTM-3p* by 4h IAA treatment. Group 3 are genes induced in *STTM-5p* and *IAA30-OE* by 4h IAA treatment. Group 4 are genes repressed in *STTM-5p*, *STTM-3p*, *IAA30-OE* and WT by 4h IAA treatment.

(F) GO enrichment analysis of genes induced by IAA in *STTM-5p*, *STTM-3p*, *IAA30-OE* and WT.

(G) GO enrichment analysis of genes repressed by IAA in *STTM-5p*, *STTM-3p*, *IAA30-OE* and WT.

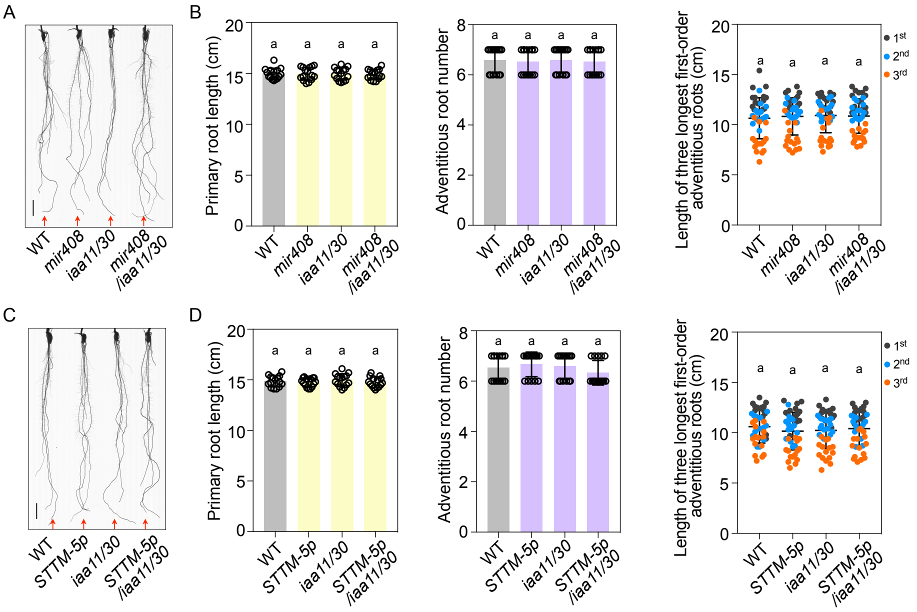

Supplementary Figure 12 The root performances of *mir408* and *STTM-5p* in *iaa11/30* background with or without IAA treatment

(A) Representative phenotype of seedling roots of 2-week-old WT, *mir408*, *iaa11/iaa30* and *mir408/iaa11/30* plants without auxin treatment. Bar = 1cm

(B) Primary root length, adventitious root number and the length of three longest first-order crown roots in 2-week-old WT, *mir408*, *iaa11/iaa30* and *mir408/iaa11/30* plants without auxin treatment.

(C) Representative phenotype of seedling roots of 2-week-old WT, *STTM-5p*, *iaa11/iaa30* and *STTM-5p/iaa11/30* plants without auxin treatment. Bar = 1cm

(D) Primary root length, adventitious root number and the length of three longest first-order crown roots in 2-week-old WT, *STTM-5p*, *iaa11/iaa30* and *STTM-5p/iaa11/30* plants without auxin treatment. Source data underlying Supplementary Fig. 12B and D are provided as a Source Data file.

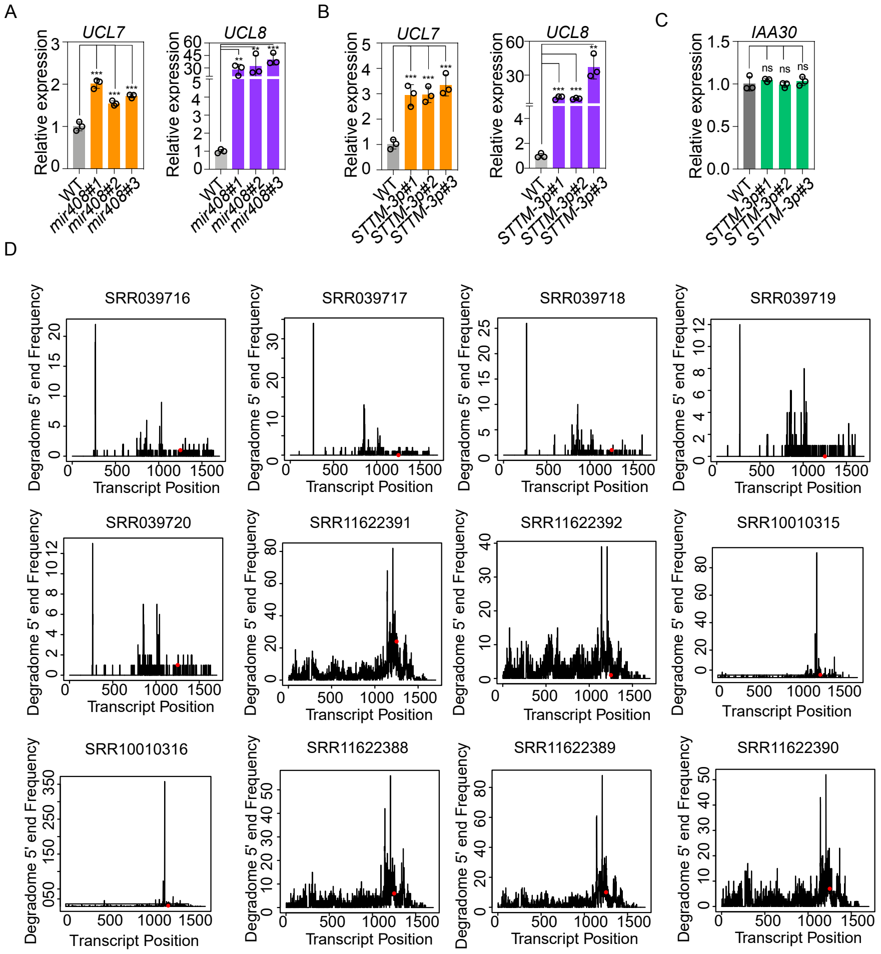

Supplementary Figure 13 Regulation of *IAA30* by miR408-5p is not in an mRNA cleavage manner under normal conditions

(A) qRT-PCR analysis of *UCL7* and *UCL8* expressions in WT and *mir408* mutants. Error bars represent SD for three replicates. Statistical analysis was performed by using Multiple comparisons test (**P < 0.01,***P < 0.001)

(B) qRT-PCR analysis of *UCL7* and *UCL8* expressions in WT and *STTM-3p* transgenic plants.

(C) qRT-PCR analysis of *IAA30* expressions in WT and *STTM-3p* transgenic plants.

(D) Distribution of the cleavage tags along the full-length of the *OsIAA30* mRNA sequence in the published degradome datasets. The red dot represents the sliced target transcripts. The sample that has more than 20 reads mapped to *OsIAA30* mRNA sequence was used for degradome analysis. Source data underlying Supplementary Fig. 13A-C are provided as a Source Data file.

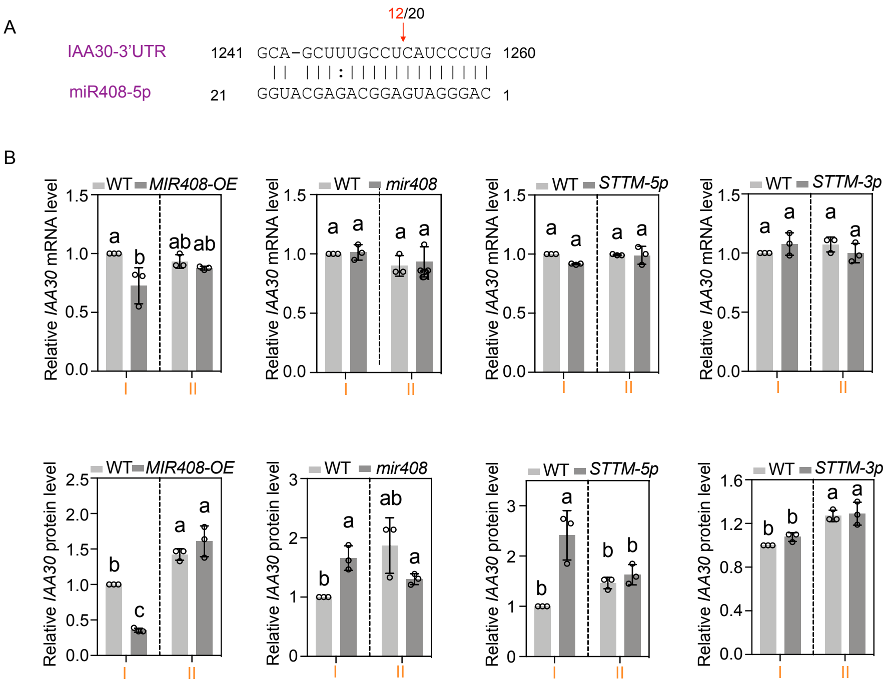

Supplementary Figure 14 miR408-5p mediates the regulation of *IAA30* through translation repression under normal conditions, but switches to mRNA digestion if it is overproduced

(A) 5' RACE analysis of *IAA30* mRNA cleavage by miR408-5p in *MIR408-OE* plants. The frequency of the sequenced 5' ends is plotted against the position in the *IAA30* target site.

(B) Relative mRNA and protein abundance of IAA30 when normal *IAA30 3'UTR* (Ⅰ) or mutated *IAA30 3'UTR* (*mUTR*) (Ⅱ) construct shown in Figure 4F was introduced into the protoplasts from *MIR408-OE*, *mir408*, *STTM-5p* and *STTM-3p* plants. Source data underlying Supplementary Fig. 14B are provided as a Source Data file.

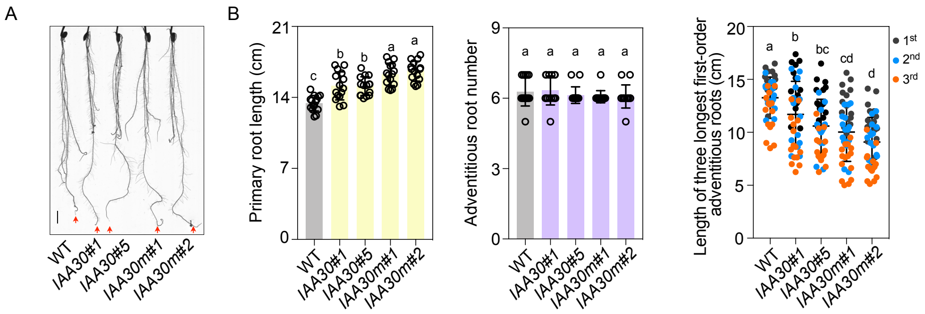

Supplementary Figure 15 The root development of *IAA30* and *IAA30m* transgenic plants without auxin treatment.

(A) Representative phenotype of seedling roots of 2-week-old WT, *IAA30* and *IAA30m* plants without IAA treatment. Bar = 1cm

(B) Primary root length, adventitious root number and the length of three longest first-order crown roots in 2-week-old WT, *IAA30* and *IAA30m* plants without IAA treatment. The different letters on top of each bar denote significant differences (Tukey's honestly significant difference, P < 0.05). Source data underlying Supplementary Fig. 15B are provided as a Source Data file.

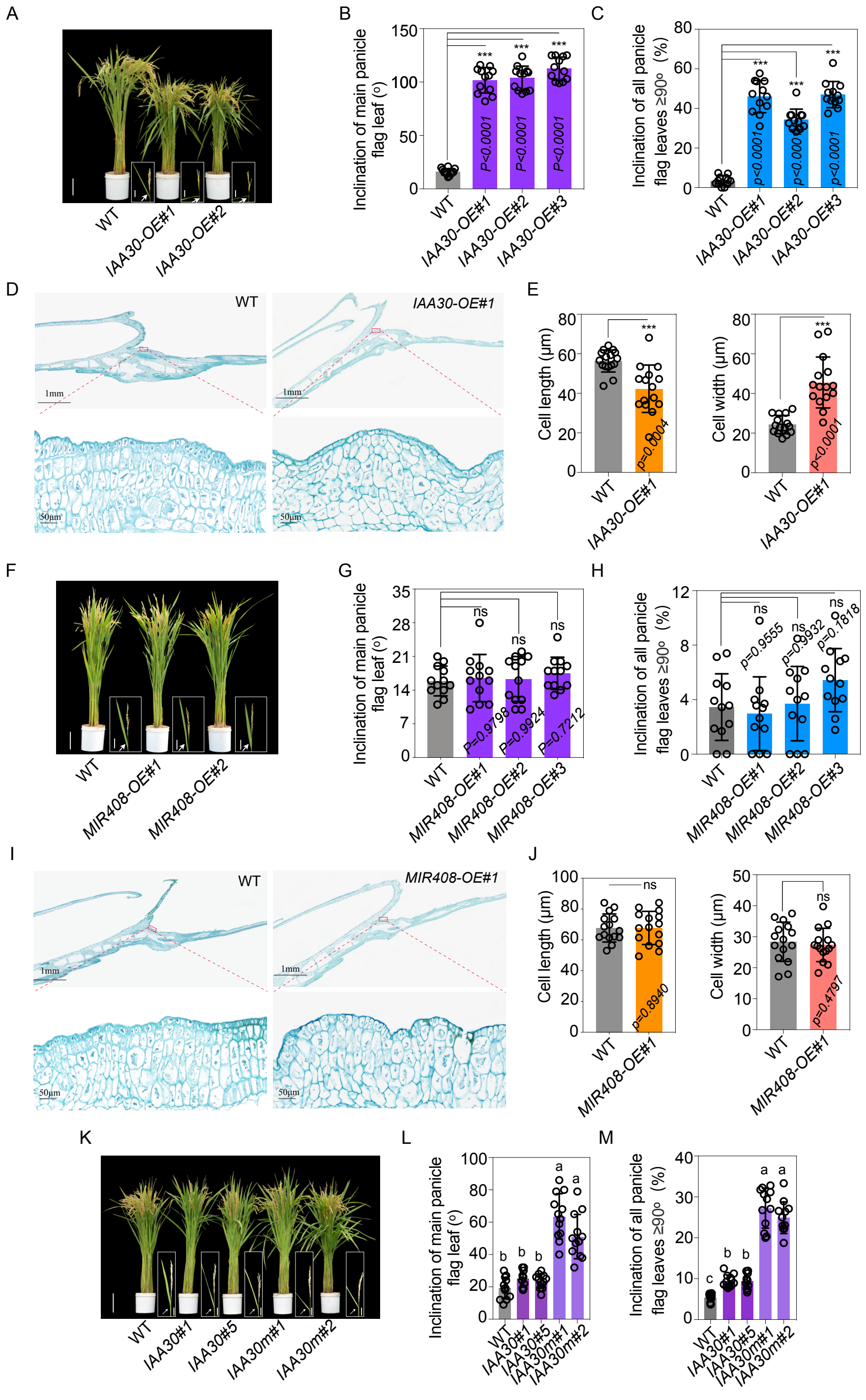

Supplementary Figure 16 IAA30 regulates leaf inclination in rice

(A) Phenotypic observations of WT and *IAA30-OE* plants after heading (Bar = 10cm). Boxed regions are enlarged to the performance of flag leaves with panicles (Bars = 1cm). White arrows indicate the lamina joints.

(B) Inclination of flag leaf on main stem with panicle in WT and *IAA30-OE* plants. Angles of flag leaf at 40 days after heading were measured and data are presented as means ± SD (standard

deviation, n = 12).

(C) Ratio of flag leaves with angles ≥ 90° from all flag leaves on stems with panicles in WT and *IAA30-OE* plants.

(D) Longitudinal section of the adaxial region of the lamina joint in WT and *IAA30-OE* flag leaves at 40 days after heading. The marked regions by red color were magnified to highlight the differences.

(E) Cell length and cell width of adaxial parenchyma cells of lamina joint in WT and *IAA30-OE* plants.

(F) Phenotypic observations of WT and *MIR408-OE* plants after heading (Bar = 10cm). Boxed regions are enlarged to the performance of flag leaves with panicles (Bars = 1cm).

(G) Inclination of flag leaf on main stem with panicles in WT and *MIR408-OE* plants.

(H) Ratio of flag leaves with angles ≥ 90° from all flag leaves on stems with panicles in WT and *MIR408-OE* plants.

(I) Longitudinal section of the adaxial region of the lamina joint in WT and *MIR408-OE* flag leaves at 40 days after heading. The marked regions by red color were magnified to highlight the differences.

(J) Cell length and cell width of adaxial parenchyma cells of lamina joint in WT and *MIR408-OE* plants.

(K) Phenotypic observations of WT, *IAA30* and *IAA30m* plants after heading (Bar = 10cm). Boxed regions are enlarged to the performance of flag leaves with panicles (Bars = 1cm). White arrows indicate the lamina joints.

(L) Inclination of flag leaf on main stem with panicles in WT, *IAA30* and *IAA30m* plants.

(M) Ratio of flag leaves with angles ≥ 90° from all flag leaves on stems with panicles in WT, *IAA30* and *IAA30m* plants. Source data underlying Supplementary Fig. 16B, C, E, G, H, J, L, and M are provided as a Source Data file.

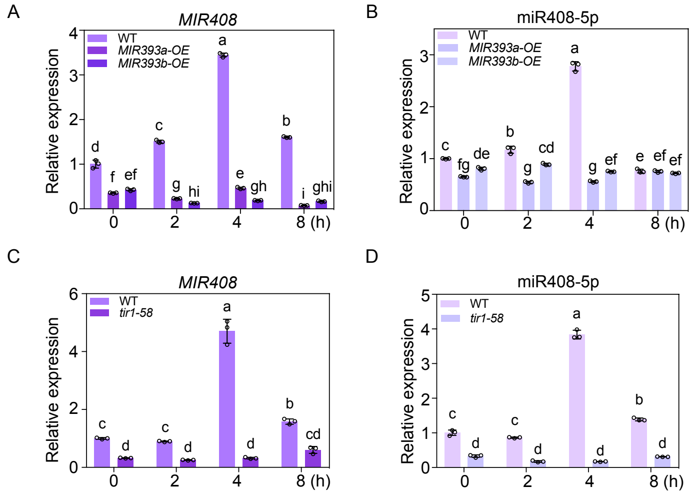

Supplementary Figure 17 miR408-5p is regulated by miR393-TIR1/AFBs module in response to auxin treatment

(A) and (B) Relative expressions of *MIR408* (A) and miR408-5p (B) in WT and miR393 overexpression (*MIR393a-OE* and *MIR393b-OE*) with the indicated time of 10μM IAA treatment.

(C) and (D) Relative expressions of *MIR408* (C) and miR408-5p (D) in WT and *tir1* mutants with the indicated time of 10μM IAA treatment. Source data underlying Supplementary Fig. 17A-D are provided as a Source Data file.

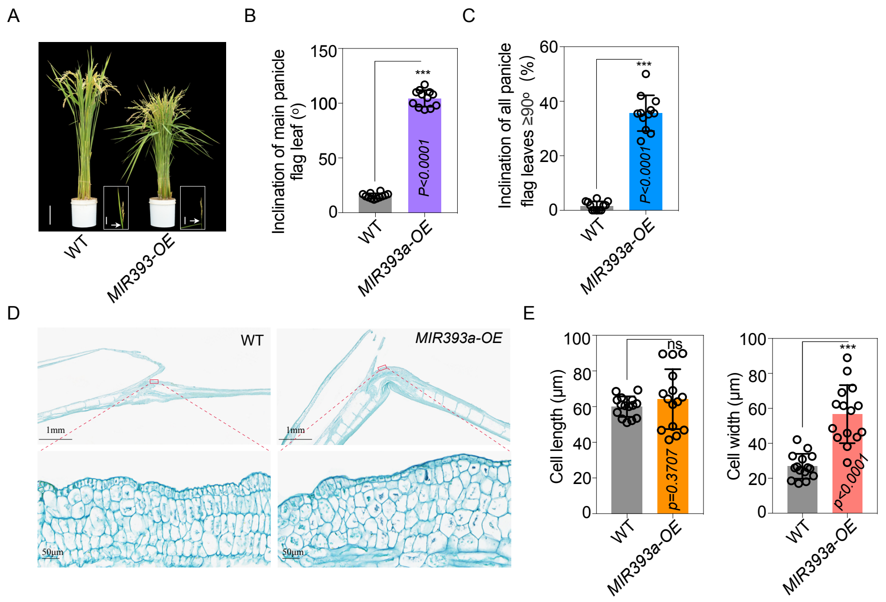

Supplementary Figure 18 miR393-TIR1/AFBs module regulates leaf inclination in rice

(A) Phenotypic observations of WT and *MIR393a-OE* plants after heading (Bar = 10cm). Boxed regions are enlarged to the performance of flag leaves with panicles (Bars = 1cm). White arrows indicate the lamina joints.

(B) Inclination of flag leaf on main stem with panicle in WT and *MIR393a-OE* plants. Angles of flag leaf at 40 days after heading were measured and data are presented as means ± SD. Statistical analysis was performed by Multiple comparisons test (***P < 0.001)

(C) Ratio of flag leaves with angles ≥ 90° from all flag leaves on stems with panicles in WT and *MIR393a-OE* plants.

(D) Longitudinal section of the adaxial region of the lamina joint in WT and *MIR393a-OE* flag leaves at 40 days after heading. The marked regions by red color were magnified to highlight the differences.

(E) Cell length and cell width of adaxial parenchyma cells of lamina joint in WT and *MIR393a-OE* plants. Source data underlying Supplementary Fig. 18B, C, and E are provided as a Source Data file.

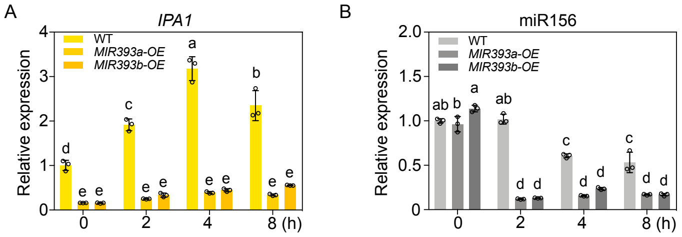

Supplementary Figure 19 The effects of miR393-TIR1/AFBs module on miR156 and *IPA1* expression in response to auxin treatment

(A) Relative expressions of *IPA1* in WT, *MIR393a-OE* and *MIR393b-OE* plants with the indicated time of 10μM IAA treatment.

(B) Relative accumulations of miR156 in WT, *MIR393a-OE* and *MIR393b-OE* plants with the indicated time of 10μM IAA treatment. Source data underlying Supplementary Fig. 19A and B are provided as a Source Data file.
