# Supplementary material for: Switching action modes of miR408-5p mediates auxin signaling in rice
Source: Nat Commun. 2024 Mar 21;15:2525. doi: 10.1038/s41467-024-46765-z (PMC10958043; doi:10.1038/s41467-024-46765-z)
Supplement: Supplementary file 3 — Description of Additional Supplementary Files [file 41467_2024_46765_MOESM3_ESM.pdf]

<u>Description of Additional Supplementary Files</u>

Supplementary Data 1 : The detailed list of auxin responsive genes in WT, *STTM-3p*, *STTM- 5p* and *IAA30-OE*

Supplementary Data 2 : Clustering analysis of expression pattern for genes responsive to auxin in WT, *STTM-3p*, *STTM-5p* and *IAA30-OE*

Supplementary Data 3 : Oligos used in this study
