# Supplementary material for: Switching action modes of miR408-5p mediates auxin signaling in rice
Source: Nat Commun. 2024 Mar 21;15:2525. doi: 10.1038/s41467-024-46765-z (PMC10958043; doi:10.1038/s41467-024-46765-z)
Supplement: Supplementary file 7 — Reporting Summary [file 41467_2024_46765_MOESM7_ESM.pdf]

nature portfolio

Corresponding author(s): Liang Wu

Last updated by author(s): Mar 4, 2024

Reporting Summary

Nature Portfolio wishes to improve the reproducibility of the work that we publish. This form provides structure for consistency and transparency in reporting. For further information on Nature Portfolio policies, see our Editorial Policies and the Editorial Policy Checklist.

Please do not complete any field with "not applicable" or n/a. Refer to the help text for what text to use if an item is not relevant to your study.
For final submission: please carefully check your responses for accuracy; you will not be able to make changes later.

Statistics

For all statistical analyses, confirm that the following items are present in the figure legend, table legend, main text, or Methods section.

n/a	Confirmed	
☐	☒	The exact sample size (*n*) for each experimental group/condition, given as a discrete number and unit of measurement
☐	☒	A statement on whether measurements were taken from distinct samples or whether the same sample was measured repeatedly
☐	☒	The statistical test(s) used AND whether they are one- or two-sided<br>*Only common tests should be described solely by name; describe more complex techniques in the Methods section.*
☒	☐	A description of all covariates tested
☒	☐	A description of any assumptions or corrections, such as tests of normality and adjustment for multiple comparisons
☐	☒	A full description of the statistical parameters including central tendency (e.g. means) or other basic estimates (e.g. regression coefficient) AND variation (e.g. standard deviation) or associated estimates of uncertainty (e.g. confidence intervals)
☐	☒	For null hypothesis testing, the test statistic (e.g. *F*, *t*, *r*) with confidence intervals, effect sizes, degrees of freedom and *P* value noted<br>*Give P values as exact values whenever suitable.*
☒	☐	For Bayesian analysis, information on the choice of priors and Markov chain Monte Carlo settings
☒	☐	For hierarchical and complex designs, identification of the appropriate level for tests and full reporting of outcomes
☒	☐	Estimates of effect sizes (e.g. Cohen's *d*, Pearson's *r*), indicating how they were calculated

Our web collection on statistics for biologists contains articles on many of the points above.

Software and code

Policy information about availability of computer code

Data collection	Degradome raw data were generated by Illumina HiSeq 2500 (LC-Bio, China); RNA-seq raw data were generated by sequencing Illumina HiSeq 2500 (LC Sciences, Hangzhou, China); Figures of Luciferase and Western Blot were generated by Tanon 5200(China); RT-qPCR raw data were generated by SteponePlus TM System (Thermo-Fisher); Cell length and width data were calculated by Image J softwarel.8.0(NIH,USA).
Data analysis	Degradome reads were matched to generate the degradome density file using script Cleaveland3_map2dd.pl; RNA-seq raw reads were further processed withHisat2, StringTie (version 2.0) and Ballgown (version 2.16.0); GO functional enrichment was analyzed by AgriGO v2.0; Significant different tests were carried out by Graph Pad Prism 8.0 (GraphPad, USA) and Microsoft office Excel (Microsoft, USA).

For manuscripts utilizing custom algorithms or software that are central to the research but not yet described in published literature, software must be made available to editors and reviewers. We strongly encourage code deposition in a community repository (e.g. GitHub). See the Nature Portfolio guidelines for submitting code & software for further information.

Data

Policy information about availability of data

All manuscripts must include a data availability statement. This statement should provide the following information, where applicable:

- Accession codes, unique identifiers, or web links for publicly available datasets
- A description of any restrictions on data availability
- For clinical datasets or third party data, please ensure that the statement adheres to our policy

source data are provided in this paper.

Research involving human participants, their data, or biological material

Policy information about studies with human participants or human data. See also policy information about sex, gender (identity/presentation), and sexual orientation and race, ethnicity and racism.

Reporting on sex and gender	N/A
Reporting on race, ethnicity, or other socially relevant groupings	N/A
Population characteristics	N/A
Recruitment	N/A
Ethics oversight	N/A

Note that full information on the approval of the study protocol must also be provided in the manuscript.

Field-specific reporting

Please select the one below that is the best fit for your research. If you are not sure, read the appropriate sections before making your selection.

☒ Life sciences ☐ Behavioural & social sciences ☐ Ecological, evolutionary & environmental sciences

For a reference copy of the document with all sections, see nature.com/documents/nr-reporting-summary-flat.pdf

Life sciences study design

All studies must disclose on these points even when the disclosure is negative.

Sample size	The sample sizes in our study were chosen based on similar experiments in relevant publications in this field to allow for confident statistical analysis. For Degradome Sequencing assays, at least 0.5 g the indicated materials were harvested as one sample. For RNA-seq, RT-PCR and qPCR, at least 0.2 g root were harvested for RNA extraction and pooled as one sample. For transfection of protoplasts assays, at least 100 seedling plants were harvested per sample. For phenotyping, at least 10 plants were measured. These all fulfill a sufficient reproducibility. Each experiment was repeated three times with similar results.
Data exclusions	No data were excluded.
Replication	Each experiment was reproduced and repeated at least three times. The information on replication has been included in all relevant figure legends.
Randomization	For each experiment, different plant genotypes were allocated randomly in the growth chambers or field without any bias.
Blinding	No blinding. Because the experimental materials are plants, blinding is not common practice for this type of experiments.

Reporting for specific materials, systems and methods

We require information from authors about some types of materials, experimental systems and methods used in many studies. Here, indicate whether each material, system or method listed is relevant to your study. If you are not sure if a list item applies to your research, read the appropriate section before selecting a response.

Materials & experimental systems

n/a	Involved in the study
☐	☒ Antibodies
☒	☐ Eukaryotic cell lines
☒	☐ Palaeontology and archaeology
☒	☐ Animals and other organisms
☒	☐ Clinical data
☒	☐ Dual use research of concern
☐	☒ Plants

Methods

n/a	Involved in the study
☒	☐ ChIP-seq
☒	☐ Flow cytometry
☒	☐ MRI-based neuroimaging

Antibodies

Antibodies used	Mouse monoclonal anti-Flag(Sigma-Aldrich,Cat#F3165),Rabbit polyclonal anti-Actin(Sangon Biotech,Cat# D110007-0100),Mouse monoclonal anti-GFP(Abclonal,Cat#AE012).
Validation	Relevant information of antibodies used in this study is found here<br>1. https://www.sigmaa ld rich.com/H K/zh/prod uct/sigma/f3165<br>2. https://www.sangon.com/productDetail?productInfo.code=D110007<br>3. https://abclonal.com.cn/catalog/ AE012

Dual use research of concern

Policy information about dual use research of concern

Hazards

Could the accidental, deliberate or reckless misuse of agents or technologies generated in the work, or the application of information presented in the manuscript, pose a threat to:

No	Yes
☒	☐ Public health
☒	☐ National security
☒	☐ Crops and/or livestock
☒	☐ Ecosystems
☒	☐ Any other significant area

Experiments of concern

Does the work involve any of these experiments of concern:

No	Yes
☒	☐ Demonstrate how to render a vaccine ineffective
☒	☐ Confer resistance to therapeutically useful antibiotics or antiviral agents
☒	☐ Enhance the virulence of a pathogen or render a nonpathogen virulent
☒	☐ Increase transmissibility of a pathogen
☒	☐ Alter the host range of a pathogen
☒	☐ Enable evasion of diagnostic/detection modalities
☒	☐ Enable the weaponization of a biological agent or toxin
☒	☐ Any other potentially harmful combination of experiments and agents

Plants

Seed stocks	The miR156 and IPA1 related rice seeds were shared from Jiayang Li, Zuhua He and Donglei Yang, other plant materials are produced from our laboratory and all seeds are stored in this laboratory.
Novel plant genotypes	All transgenic transformations were performed by Agrobacterium tumefaciens strain AGL1.The mir408, iaa30 and iaa11/30 mutants in rice were generated by CRISPR-cas9 strategy.All transgenic plants analyzed in our study were T3 generation homozygsous plants.
Authentication	All overexpression materials were identified by RT, qRT-PCR and hygromycin resistance gene. All gene editing materials were identified by sequencing. If it is necessary to identify again, the primer sequences provided in the paper will be performed by RT, qRT-PCR and sequencing.

This checklist template is licensed under a Creative Commons Attribution 4.0 International License, which permits use, sharing, adaptation, distribution and reproduction in any medium or format, as long as you give appropriate credit to the original author(s) and the source, provide a link to the Creative Commons license, and indicate if changes were made. The images or other third party material in this article are included in the article's Creative Commons license, unless indicated otherwise in a credit line to the material. If material is not included in the article's Creative Commons license and your intended use is not permitted by statutory regulation or exceeds the permitted use, you will need to obtain permission directly from the copyright holder. To view a copy of this license, visit http://creativecommons.org/licenses/by/4.0/
